# Conceptual development of an intensive exercise program for glioma patients (ActiNO): summary of clinical experience

**DOI:** 10.1007/s11060-023-04354-y

**Published:** 2023-06-12

**Authors:** Johanna Jost, Michael Müther, Ralf Brandt, Ugur Altuner, Lars Lemcke, Walter Stummer, Klaus Völker, Rainer Wiewrodt, Dorothee Wiewrodt

**Affiliations:** 1 Department of Neurosurgery, University Hospital, University Münster, Münster, Germany; 2 Department of Medicine D, University Hospital, University Münster, Münster, Germany; 3 Institute of Sports Science, University Hospital, University Münster, Münster, Germany; 4 Pulmonary Research Division, Department of Medicine A, University Hospital, University, Münster, Germany

**Keywords:** Brain tumor, Glioblastoma, High-intensity training, Ergometry, Exercise

## Abstract

**Purpose:**

Exercise proved to reduce cancer-related symptoms and prolong survival in some cancer types. However, brain tumor patients are often advised against strenuous exercise. Here, we summarize our experience with a submaximal exercise program for glioma patients: ActiNO (Active in Neuro-Oncology).

**Methods:**

Glioma patients were invited to participate in the program. Since 2011, a sports scientist individualized two one-hour sessions per week adapted to the patients’ symptoms. One session consisted of bicycle ergometry (average workload: 75% of maximum heart rate), the other of whole-body resistance training. Both sessions were further complimented by coordinative elements. Cardiorespiratory fitness was assessed using the ”Physical Work Capacity” procedure. Patients were followed up regularly to assess adherence to the program and disease activity.

**Results:**

Until December 2019, 45 glioma patients, median-aged 49 years (IQR 42–59), were included in the analysis. Most patients suffered from glioblastoma (58%), followed by diffuse lower-grade astrocytoma (29%). In overall 1828 training sessions, two minor epileptic events occurred (1 speech arrest; 1 focal seizure). During fitness assessment, all patients achieved at least 75% of their age-adjusted maximum heart rate. Peak workload averaged 172 W (95% CI 156–187). Median survival of participating glioblastoma patients was 24.1 months (95% CI 8.6–39.5).

**Conclusion:**

This supervised training program with submaximal exertion was feasible and safe in glioma regardless of WHO grading. Based on these experiences, we initiated a prospective multicenter study to objectify improvements in physical performance and quality of life in patients with glioblastoma.

**Supplementary Information:**

The online version contains supplementary material available at 10.1007/s11060-023-04354-y.

## Introduction

Despite multimodal therapies, malignant gliomas still have a poor prognosis [1]. Many glioma patients suffer from disease- or therapy-induced neurocognitive, functional, and emotional deficits, which significantly limit quality of life [2]. The main goal of all treatment efforts is therefore a prolongation of life at the highest possible quality. To reach that goal, supportive therapies are well perceived [3].

Out of many suitable supportive therapies, sportive activities and physical training for cancer patients are considered ideal for cancer patients at large. In almost 700 clinical trials with more than 50,000 cancer patients examined at different treatment time points, many positive effects of exercise, especially regarding side effect management including fatigue, polyneuropathies, psychological distress, and physical constraints have been demonstrated [3]. However, most intervention studies were conducted in patients with breast, prostate, and colon cancer. In these entities, physical training is safe, feasible, and potentially beneficial regarding patients’ physical, emotional, and cognitive well-being [4–6].

In contrast, little is known about the benefit to glioma patients [7]. In a systematic review by Sandler et al. comprising 15 studies, it was shown that the majority of patients are not sufficiently physically active after being diagnosed with a brain tumor, although more active patients showed improved quality of life as well as less severe symptom burden [8]. Given that unmet need, a personal training program for brain tumor patients was initiated at our institution. Here, we describe the setup for an intensive supervised exercise program, demonstrate its feasibility and safety, and analyze outcomes.

## Conceptual development of the exercise program

At start, the personal training program was rather unsystematically and was shaped by the participants’ individual wishes and ideas. General recommendations for cancer patients at that time, i.e., published by the American College of Sports Medicine, were considered [9]. As they focused on breast, prostate, hematologic, colorectal, and gynecologic cancers, but not on brain tumor patients, it was decided that the exercise program for multimodal treated glioma patients should be individualized. Regarding training modalities and intensity, it should be optimally conducted twice per week for 1 hour and should contain both strength and endurance elements in order to be able to achieve noticeable increases in performance. Furthermore, a special focus was placed on the integration of coordinative elements. In general, exercise tasks were adapted to the patients’ respective symptoms. If possible, exercise levels were enhanced over time. Heart rate was monitored throughout the entire session (via Polar M430 incl. HR sensor H7/9, Kempele, Finland).

**Fig. 1:**
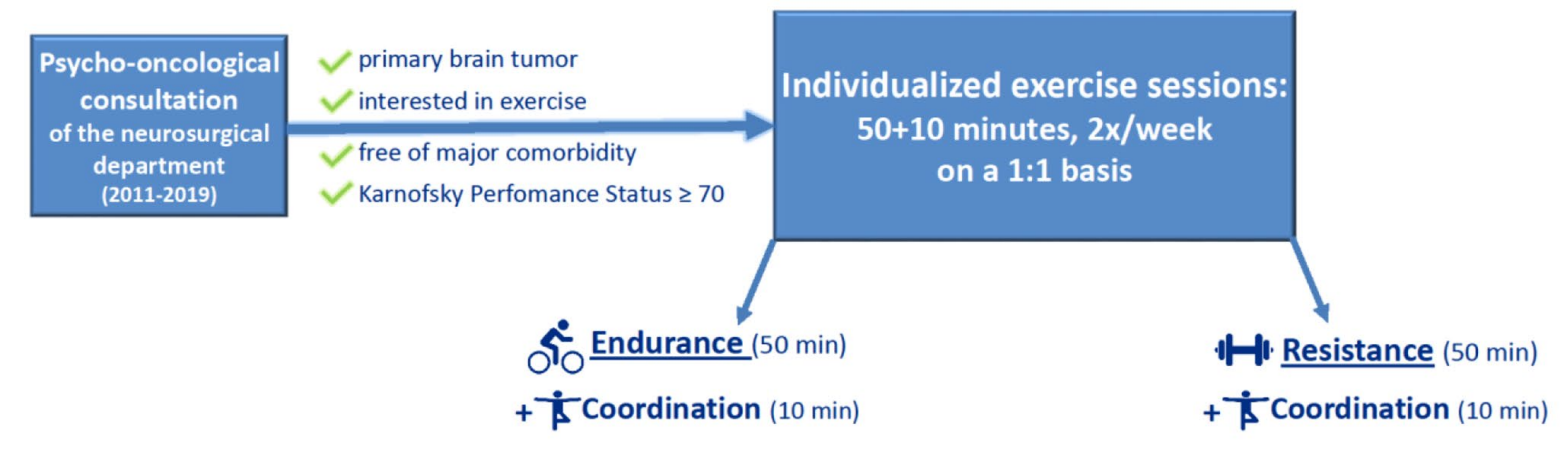
Suitable recruitment criteria and exercise modalities. The three training sections have been proven to be feasible and are adjustable to individual patients’ needs. For further information, see Fig. 2 and Supplementary Figures S1 – S3.

As the number of patients increased, the training became more systematic. We concluded that training should ideally be conducted on a 1:1-basis. Usually, each session was divided into approximately 50 min of either resistance or endurance training and completed by 10 min of coordination training (Fig. 1).

### Endurance training

**Fig. 2:**
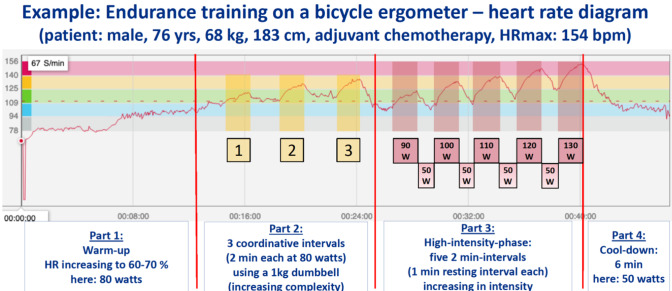
Illustration of a typical endurance training sequence (part 1 to part 4). Numbers on abscissa indicate time in minutes, ordinate reflects heart rate per minute, measured with Polar M430 and HR sensor H7/9. After warm-up, the patient is exposed to different interventions in addition to ergometric cycling (part 2). To visualize the coordinative elements during part 2, a series of dumbbell movements is shown in Supplementary Figure S1. To achieve submaximal loading, a high-intensity interval training (part 3) is performed prior to the cool-down phase (part 4). Upon completion of endurance training, approx. 10 min of coordination training (i.e. Hoop pyramid (Fig S2) or arm skill training (Fig. S3)) sums up to 60 min of training.

Endurance training is performed on a bicycle ergometer and divided into 4 parts. Due to having 5 fix points to the anti-tilt bicycle ergometer (feet, hands and bottom), even patients with balance disorders feel safe and can be loaded properly. Adjustments to workload are controlled by heart rate. Continuous speed of rotation is 70–75 rpm. After a 10-min warm-up, patients achieve 60–65% of their individual maximum heart rate (HRmax). In the next 12-min-part, 3 intervals of coordinative elements using 1–2 kg-dumbbells are integrated, workload remains constant (Suppl. Figure S1). This way, fix points are reduced to 3 (feet and bottom) and the patients’ trunk muscles are activated to compensate for any imbalances. Heart rate meanwhile increases up to 75% of HRmax. This is followed by five 2-min-intervals (1-min resting interval each), increasing in intensity (last interval at about 90% of HRmax). The training concludes with a 6-min cool-down. An average workload of 75% of HRmax throughout the session is targeted (Fig. 2).

### Resistance training

Progressive whole-body resistance training is based on up to 12 separate, but standardized exercise tasks (leg press, knee extension + flexion, adductors, abductors, butterfly + reverse, chest press, biceps + triceps curls, latissimus pulldown, abdominal machine). 3 sets of 21 repetitions each are performed. Rate of perceived exertion (RPE, scale 1–20) is at 14 when entering the program. As training progresses, intensity reaches RPE 17–20. Rests between sets last 30–60 s. A special feature of the exercise execution is a change in movement amplitude in the course of 21 repetitions. The first 7 repetitions are performed with maximum amplitude, the following 7 with half amplitude and the last 7 are small final contractions. This method is based on electromyography analyses according to Boeckh-Behrens & Buskies and aims at maximal muscle activation [10]. Execution is performed rather quickly, so that each series lasts 30–40 s. Total time under tension is 90–120 s.

### Coordination training

Coordination training consists of either arm skill training or exercises including agility hoops arranged as a pyramid. Arm skill training is based on a complex choreography including bending, stretching, and rotation tasks to both improve arm control and challenge the brain. During hoop training, patients have to cross a hoop pyramid by following a given sequence of steps, increasing in complexity (e.g., starting with 2 contacts per hoop). Further explanations on coordination training can be taken from the Supplement.

## Methods

### Study design and setting

This is a retrospective observational study in a single tertiary academic center setting. As part of the psycho-oncological consultation after surgical resection, patients at the brain tumor center of the University Hospital Münster, who were found to be suitable (interested in exercise, no severe neurologic or cognitive deficits precluding patient participation, no refractory epileptic seizures (> 3 focal seizures per day or > 1 generalized seizure within 3 days of evaluation), ECOG 0–1, age ≥ 18), were offered participation in a free personal training program. Data were extracted from the patients’ charts. Beside notion of standard baseline characteristics, adverse events during training were rated according to the Common Terminology Criteria for Adverse Events (CTCAE), version 5.0. For reporting the “Strengthening the Reporting of Observational Studies in Epidemiology (STROBE) Guidelines” were adapted. Ethical approval was obtained from the regional ethical committee (2020-296-f-S).

#### Data sources and measurements

We analyze all glioma patients that took part in the program described above from its initiation in 2011 until 2019. To assess cardiorespiratory fitness of all patients at the program’s start, an incremental, physician-monitored exercise test with continuous heart rate monitoring was performed according to guidelines published by the WHO [11]. Maximum loading was targeted to achieve the highest possible diagnostic accuracy. All tests were performed on a cycle ergometer (Ergo-Fit 3000 Cycle med, Pirmasens, Germany). Participants began cycling at 25 W. Workloads were then increased by 25 W every 2 min until volitional exhaustion. Patients were asked to maintain a speed of 70 rpm. At the end of each workload, rating of perceived exertion was evaluated using the Borg scale.

Physical fitness was determined based on heart rate data using the “Physical Work Capacity” (PWC) procedure [12, 13]. Pulse-related power in watts at 75% of age-adjusted HRmax according to Tanaka et al. [14] was calculated using linear interpolation [15]. Subsequently, the calculated PWC values were divided by the patients’ body weight to determine the achieved watts per kilogram of body weight (W/kg BW). PWC performance served as an orientation for the sports scientist to create the participants’ individual training protocol. Provided that organizational and personnel resources were available, a second cardiorespiratory exercise test was conducted to detect changes in physical fitness.

To exclude negative effects on overall survival due to intensive exercise, a survival analysis of all participating glioblastoma patients (WHO grade 4, n = 26) was introduced. For comparison, the basis for the control group were all consecutive adult patients who underwent surgery for glioblastoma at the institution between January 2011 and December 2019. Of these, the control group was carefully selected based on comparable criteria, including nutrition status (BMI), age, and received adjuvant therapies, to minimize potential selection bias. As an additional supplement to this analysis, a matched-pair analysis with all IDH-wildtype glioblastoma patients was conducted. Matching was performed based on age (with a tolerance of plus/minus 3 years), gender (exact match required), having active or previous adjuvant treatment (exact match required), MGMT status (O-6-methylguanine-DNA methyltransferase, exact match required), and BMI (with a tolerance of plus/minus 3 kg/m²).

### Statistical methods

Standard descriptive analyses were performed. Absolute and relative frequencies were used for categorical variables. Means and standard deviations (SD) were shown for normally distributed data and medians and ranges or interquartile ranges (IQRs) for data not normally distributed. Data from PWC tests were tested for normal distribution using the Shapiro-Wilk test. Mean comparison between the two test time points was then performed using either dependent samples t-test (if normally distributed) or Mann-Whitney U test (not normally distributed). Overall survival was estimated using the Kaplan-Meier method and assessed for statistical significance with the log-rank test. Data were analyzed using GraphPad Prism Version 9.0 (GraphPad Software, San Diego, CA, USA) and SPSS Version 29.0 (IBM, Armonk, NY, USA).

## Results

### Participants

Fourty-five patients (42% female), median-aged 49 years (95% CI 23–76), were included in this individual exercise program of overall 1828 training sessions. Mean BMI was 24 kg/m2 (95% CI 18–33) at the time of training commencement. Most patients suffered from glioblastoma (n = 26, 58%, referring to the WHO classification from 2016 [16]), followed by diffuse lower-grade astrocytoma (29%). The majority received subsequent treatment (Table 1). Except for one patient, all participating glioblastoma patients were undergoing adjuvant treatment at the time of training commencement and the majority of them still received concurrent radiochemotherapy (16/26, 61%).

**Table 1:** Baseline patient characteristics. Chemotherapy consisted of either temozolomide [17] or lomustine-temozolomide combination [18].

	n = 45
Male / Female (%)	26 (58) / 19 (42)
Median Body Mass Index (IQR)	24 kg/m^2^ (95% CI 18–33**)**
Diagnosis:	
Glioblastoma, WHO Grade 4 (%)	26 (58)
Diffuse Lower Grade Astrocytoma, IDH-mutated, WHO Grades 2–3 (%)	13 (29)
Other intrinsic tumor types (%)	6 (13)
Main neurological impairment at program commencement (%):	15 (33)
Motor (%)	10 (22)
Sensory (%)	1 (2)
Visual (%)	3 (7)
Coordinative (%)	1 (2)
Tumor treatment at training commencement:	
Ongoing adjuvant therapy	34 (76)
- Ongoing concomitant radiochemotherapy (%)	19 (42)
- Ongoing chemotherapy only (%)	15 (34)
No treatment at training commencement	11 (24)
Time to training commencement since last treatment (months)	5.7 (median)

### Cardiorespiratory exercise testing

**Fig. 3:**
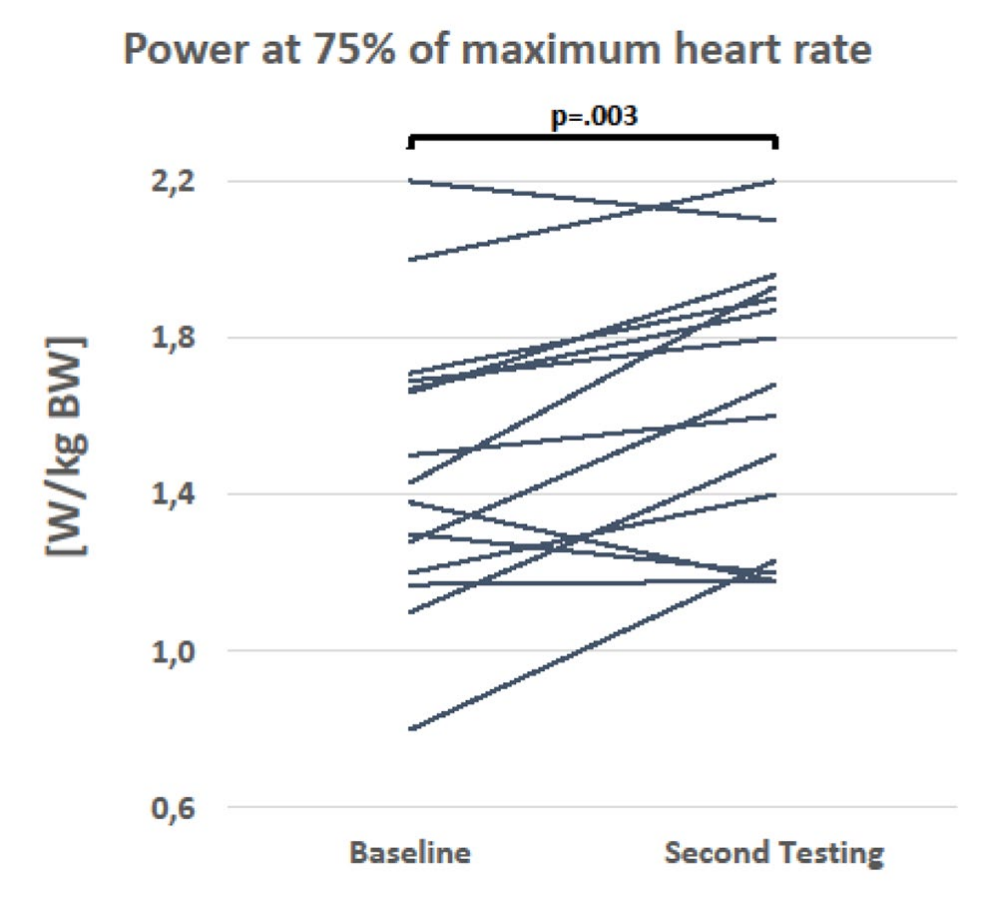
Mean scores and ranges of power at 75% of maximum heart rate (208 − 0.7 * age) in W/kg BW of patients tested twice (n = 15).

All patients managed a cardiorespiratory exercise test upon entry. At 75% of their age-adjusted HRmax, they performed an average workload of 1.46 W/kg BW (range 0.5–2.4) prior entering the training program. Female patients performed an average workload of 1.17 W/kg BW (range 0.5-2.0); the corresponding power score for male patients was 1.66 W/kg BW (range 0.7–2.4) (data not shown). During this open, voluntary exercise program, it was possible to perform a second exercise test in 15 patients (6 women). Their mean score was 1.48 W/kg BW at the beginning of the program and increased to 1.65 W/kg BW (p = 0.003), an improvement of 11.5% on average (p < 0.003, intra-individual comparison). Regarding sex, women’s average score increased from 1.25 W/kg BW (range 0.8–1.7) to 1.48 W/kg BW (range 1.2–1.9), and the score for men raised from 1.63 W/kg BW (range 1.2–2.2) to 1.77 W/kg BW (1.2–2.2). In total, eleven patients improved (73%), one patient maintained his performance, and three deteriorated (Fig. 3).

Despite the increase in workload to volitional exhaustion, no complications occurred during incremental exercise testing. Due to weakness of the legs, one patient had to stop at a perceived effort of 15. All patients achieved at least 75% of their age-adjusted heart rate. Peak workload averaged 172 W (range 50–275 W, 95% CI 156–187) and the mean perceived effort at peak was 18.7 (Borg scale, range 15–20, 95% CI 18.3–19.1).

### Adverse events during training

During 1828 training sessions, only two self-limiting seizures (focal seizure and speech arrest, both grade 1 according to CTCAE Version 5) occurred (0.1%). Both patients were familiar with the respective type of seizure and training was continued with reduced intensity. No training-related moderate or severe adverse events (grade 2–4) were observed.

### Follow-up

The patients accomplished a median of 16 (IQR 7–49) training sessions. Thirty-six (80%) patients quit the training program. The most common reason (44%) to discontinue training was tumor progression/death, followed by infrastructural reasons (i.e., distance between training site and home). Until today, more than 10 years after program initiation, 27 of the 45 (60%) patients have died. Of the 18 survivors, 15 (83%) continue exercising: 9 patients continue training on their own (counseling offered if needed), and further 6 patients exercise under occasional supervision. One patient had to stop exercising after suffering a brainstem stroke 15 month after entering the training program and two patients are lost to follow-up.

### Survival analysis for glioblastoma

**Fig. 4:**
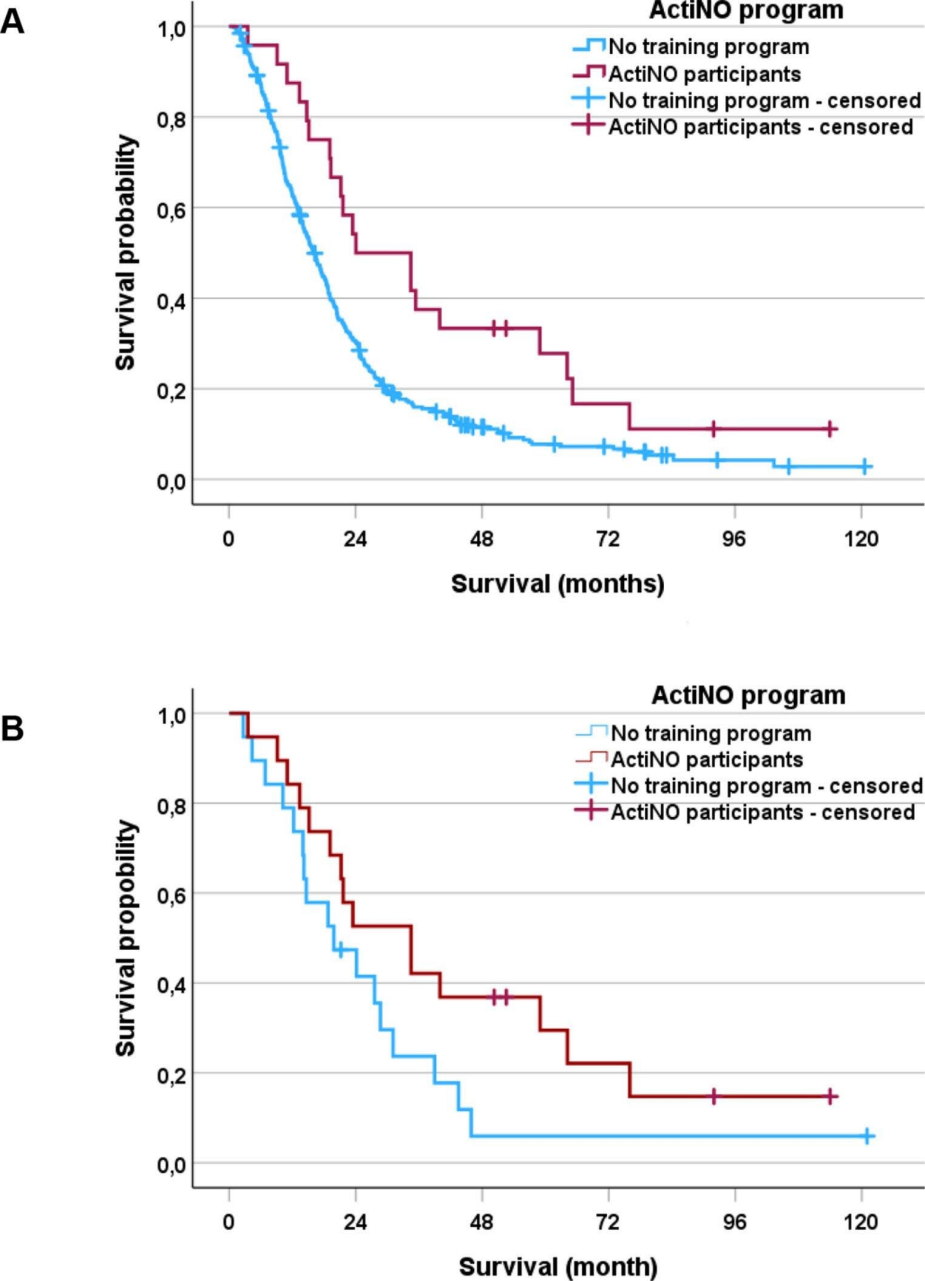
Kaplan Meier plot demonstrates a relationship between overall survival and participating in an individualized, voluntary, intensive exercise program. Figure 4 A displays a median survival of the participating patients with glioblastoma of 24.1 months (95% CI 8.6–39. 5) compared to an age, therapy-, and nutritional-selected comparator mimicking the exercise intervention group. The median survival of comparator group was 16.0 months (95% CI 14.2–17.9), p < 0.005). Figure 4B shows a matched-pair analysis in IDH-wildtype glioblastoma patients (n = 19 in each group); due to the small sample size, statistical power is limited, p = 0.099. Intensive training does not harm, however the conclusion of a survival benefit as a direct consequence of intensive exercise training cannot be drawn due to the retrospective, solely descriptive data analysis.

From 2011 to 2019, 579 glioblastoma patients were surgically treated at the University Hospitals’ Brain Tumor Center. Median survival of all consecutive adult patients was 11.8 month (95% CI 9.1–14.5; data not shown).The median survival of ActiNO participants with glioblastoma (n = 26) was 24.1 months (95% CI 8.6–39.5). To address the positive selection of the ActiNO participants (all of them received adjuvant multimodal therapy including radiochemotherapy with temozolomide [17] or a combination of lumostine and temozolomide [18], their younger age (the oldest ActiNO participant was 76 years), and their better nutrition status (the highest BMI was 32.9 kg/m2), the control cohort should have the same selection criteria (all were treated multimodally with radiochemotherapy, age ≤ 77 years, BMI ≤ 33 kg/m2). Given that, 325 out of 579 patients (56%) met these criteria. The ActiNO group and the control cohort did not significantly differ in terms of age and BMI (p > 0.05, all comparisons). The median survival of this carefully chosen control cohort (n = 325) was 16.0 month (95% CI 14.2–17.9), p < 0.005 compared to the ActiNO group) (Fig. 4A). Due to the solely descriptive character of the study, the large difference in sample size of the two groups, and the potential selection bias of the ActiNO group, further Cox proportional hazard regression analysis was not performed. The survival benefit of the ActiNO group is considered clinically meaningful but hypothesis generating only. To further strengthen this observation, and to raise the evidence that ActiNO-activities definitely did not harm, a matched-pair analysis was conducted (Fig. 4B). Although the survival curves separate quite similarly, the observed difference did not reach statistical significance (p = 0.099) due to the limited sample size. However, a clear trend favoring the ActiNO group is still discernible.

## Discussion

We report on the first series of intensified physical exercise for diffuse glioma patients including glioblastoma. Based on our experience with over 1800 training sessions in a total of 45 patients, several major conclusions can be drawn.

First, submaximal physical training is tolerable and safe in selected patients with diffuse glioma, even in high-grade gliomas, and even under adjuvant therapies. High-intensity training despite various impairments is possible. We saw no falls or other exercise-related injuries. Only two minor focal seizures (0.1% of training sessions) were noted. Seizures did not reoccur during subsequent training and patients were willing and able to continue training immediately. In a systematic review van den Bongard et al. found that exercise does not usually have a negative effect on seizure frequency in epileptics [19]. Furthermore, our experience is consistent with that of 13 studies that demonstrated safety of less intensive exercise programs in brain tumor patients [8].

Second, incremental exercise testing to volitional exhaustion under supervision is a safe and feasible procedure, which has not been demonstrated before. To the best of our knowledge, there is only one study in glioblastoma patients with maximal exercise stress during cardiorespiratory exercise testing. In contrast to our study, some of those patients were unable to achieve maximal cardiovascular function due to fatigue and leg weakness [20].

Third, and in line with other interventional studies in glioma patients [4, 20–24], we were able to demonstrate an improvement in physical performance through a structured exercise program. Although the studies cited are small samples, a clear trend can be seen for both strength parameter and cardiorespiratory fitness. Nevertheless, it should be noted that a certain level of exercise intensity might be necessary to increase physical fitness. In addition, it has been shown that the greatest benefit in quality of life in cancer patients was achieved with exercise programs of at least 120 min of activity per week, for at least 2 month, including a minimum of 15 min of high-intensity training [25]. Appropriate intervention studies testing more intensive versus less intensive training on several outcome parameters (incl. quality of life and physical functioning) in brain tumor patients are warranted. Of note, one of our glioblastoma patients managed to run a marathon and kept improving his performance despite multimodal therapy [26]. This example emphasizes that extreme exercise exposures are feasible for selected glioma patients.

Looking at the high adherence rates to exercise programs for brain tumor patients (ranging from 79 to 100%), motivation and interest in sports are seemingly high [4, 6, 22, 23]. Similar to Capozzi et al. [24], disease progression and infrastructural reasons were the most common causative factors for discontinuation of exercise. Another motivational component is the personal contact with the trainer. A systematic review has shown that supervised training is more effective than unsupervised exercise [27].

The fourth major observation is that high intensive physical exercise does not negatively impact survival in high-grade brain tumor patients. Quite opposite, patients receiving submaximal exercise training showed a trend towards prolonged survival. This has not yet been reported for glioblastoma. In other cancers, higher levels of physical activity are related to positive impacts on survival outcomes [28]. A randomized controlled trial including 242 breast cancer patients showed that exercise improved chemotherapy tolerance [29]. Furthermore, follow-up suggests a more favorable, but non-significant disease-free survival as well as overall survival for both breast cancer exercise groups [30]. In addition, in the observational study by Holmes et al. [31], breast cancer patients being more active had a reduced risk of mortality. Regular physical activity has also been shown to prolong survival in colorectal cancer [32–34]. Similarly, there are associations between lower overall mortality and cancer-specific mortality in men with prostate cancer [35].

With regard to the Kaplan-Meier analysis, a harmful effect on overall survival due to maximal physical activity can be excluded in glioblastoma patients. Moreover, despite carefully matching, and a still possible selection bias of the participants in this voluntary program, a trend towards prolonged survival is visible and hypothesis generating. Further studies have to prove possible survival benefits.

The potential mechanism to create a survival benefit for glioma patients with increasing physical fitness may be the building of higher muscle strength and muscle mass. Temporal muscle thickness of newly diagnosed glioblastoma patients at baseline as well as the extent of its loss over time, for instance, are of prognostic relevance [36]. Since temporal muscle thickness is highly correlated with muscle strength [37], it may be of particular interest to counteract general muscle wasting with appropriate interventions. Benefits from physical activity have also been shown in mouse models of glioma [38–40]. Tantillo et al., e.g., report on reduced tumor proliferation rates as well as later onset of motor deficits by voluntary physical exercise in glioma-bearing mice [40]. Importantly, in the study of Lemke et al., this prolonged survival through exercise was significant only when combined with temozolomide treatment [39].

The main limitation of our study is its retrospective character and the heterogeneity of tumor types. Potential selection biases reduce the likelihood of generalizability of the results. When comparing with the performance values of a large representative German cohort [41], the glioma patients tested here show a trend towards above average performance values at training commencement. Those patients, athletic and interested in sports, may maintain superior physical fitness even in case of severe disease, resulting in potentially favorable long-term outcome.

## Conclusion

Our data demonstrate that intensive exercise is well tolerated and performance enhancing. Despite a common fear of potential risks, we did not detect any moderate or severe adverse events in almost 2000 training sessions. To further objectify improvements in physical performance, quality of life, and cognition in patients with glioblastoma, we initiated a prospective multicenter study that is currently recruiting (ClinicalTrials.gov Identifier: NCT05015543).

## Electronic supplementary material

Below is the link to the electronic supplementary material.

Supplementary Material 1
