# Supplementary material for: Conceptual development of an intensive exercise program for glioma patients (ActiNO): summary of clinical experience
Source: J Neurooncol. 2023 Jun 12;163(2):367–76. doi: 10.1007/s11060-023-04354-y (PMC10322750; doi:10.1007/s11060-023-04354-y)
Supplement: Supplementary file 1 — Supplementary Material 1 [file 11060_2023_4354_MOESM1_ESM.pdf]

SUPPLEMENT

Article: Conceptual development of an intensive exercise program for glioma patients (ActiNO): summary of clinical experience

Journal of Neuro-Oncology

Authors: Jost J, Müther M, Brandt R, Altuner U, Stummer W, Völker K, Wiewrodt R and Wiewrodt D

Corresponding author: Johanna Jost, University Hospital Münster, Germany; email: johanna.jost@ukmuenster.de,

Please note: The patients depicted agree to the use of the photos shown here. Written consent has been obtained.

Supplementary figure 1: Example of coordinative elements during endurance training (bicycle ergometry, part 2)

Three intervals of coordinative elements using 1-2 kg-dumbbells are performed during endurance training. The first interval includes a bending task for 2 minutes. This is followed by a rest interval of 2 minutes, during which patients just swing the dumbbells. In the next interval, the patients add a stretching task to bending and perform these movements alternately for 2 minutes. After another rest (swinging), the last element, the overhead press, is added to the other two movement tasks for the final interval.

**(1) bending**	
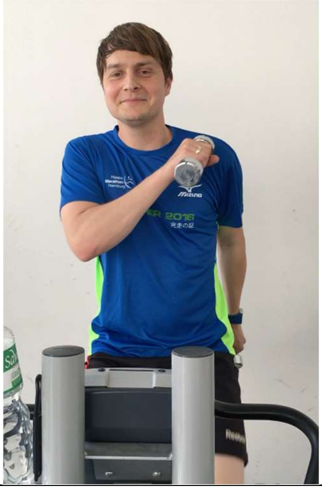	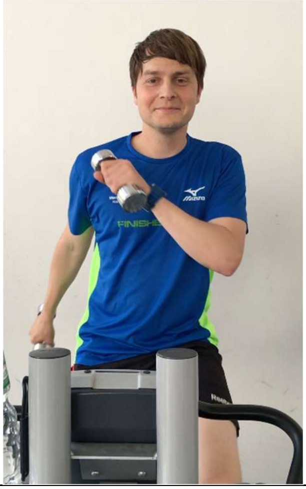
**(2) stretching**	
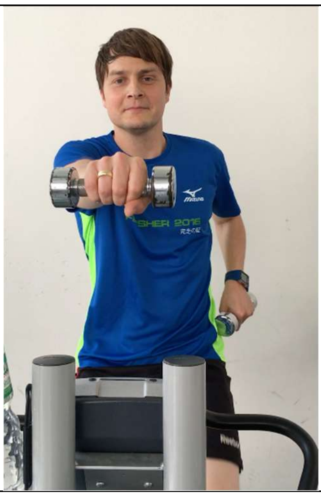	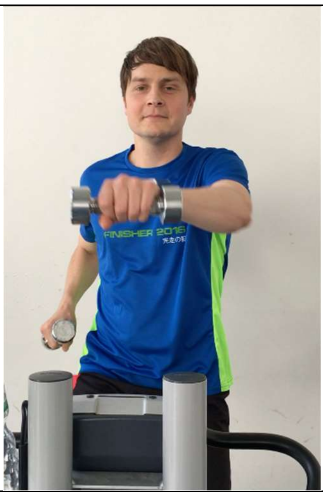
**(3) overhead press**	
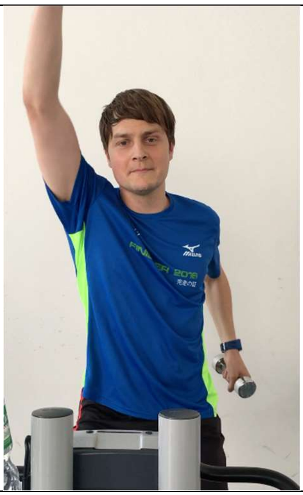	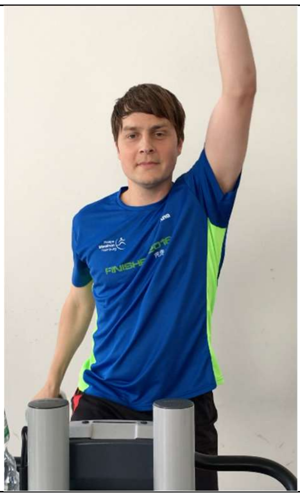

Supplementary figure 2: Coordination training with hoop pyramid

The aim is to cross the hoops following a given sequence of steps (e.g. 2/3/4 steps per hoop). Variations include stepboards, dumbbells, arms, acoustic/visual signals, clapping hands, calculation tasks, or moving sideward/backward

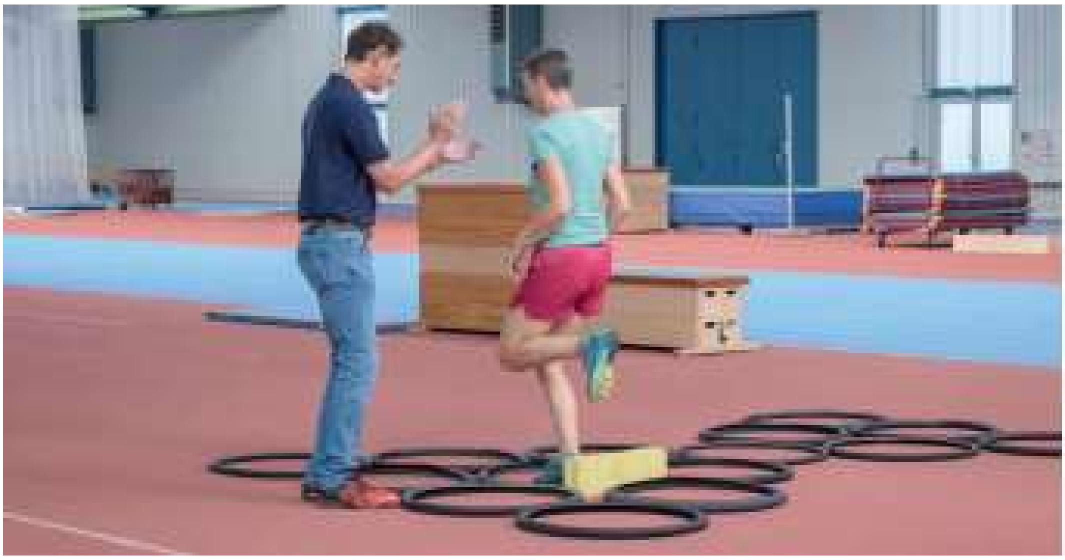

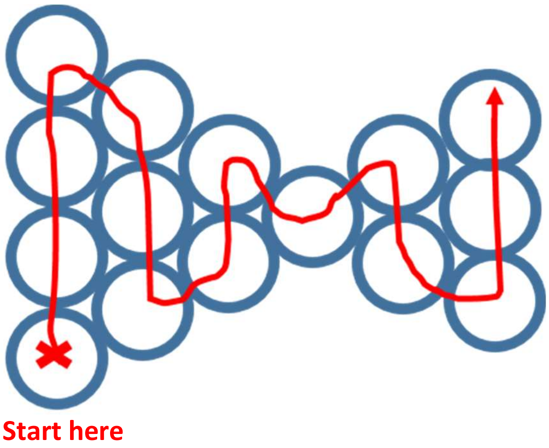

Supplementary figure 3: Coordination training with arm skill movements

To execute the movements, both dumbbell and Thera-Band are used. Here, the right arm guides the dumbbell with alternating bending, stretching, and twisting movements. Possible movement variations and combinations can be taken from the pictures.

The Thera-Band runs diagonally behind the back from the right foot to the left hand. The left upper arm is parallel to the floor. Bending and stretching movements are permanently performed in the left elbow joint.

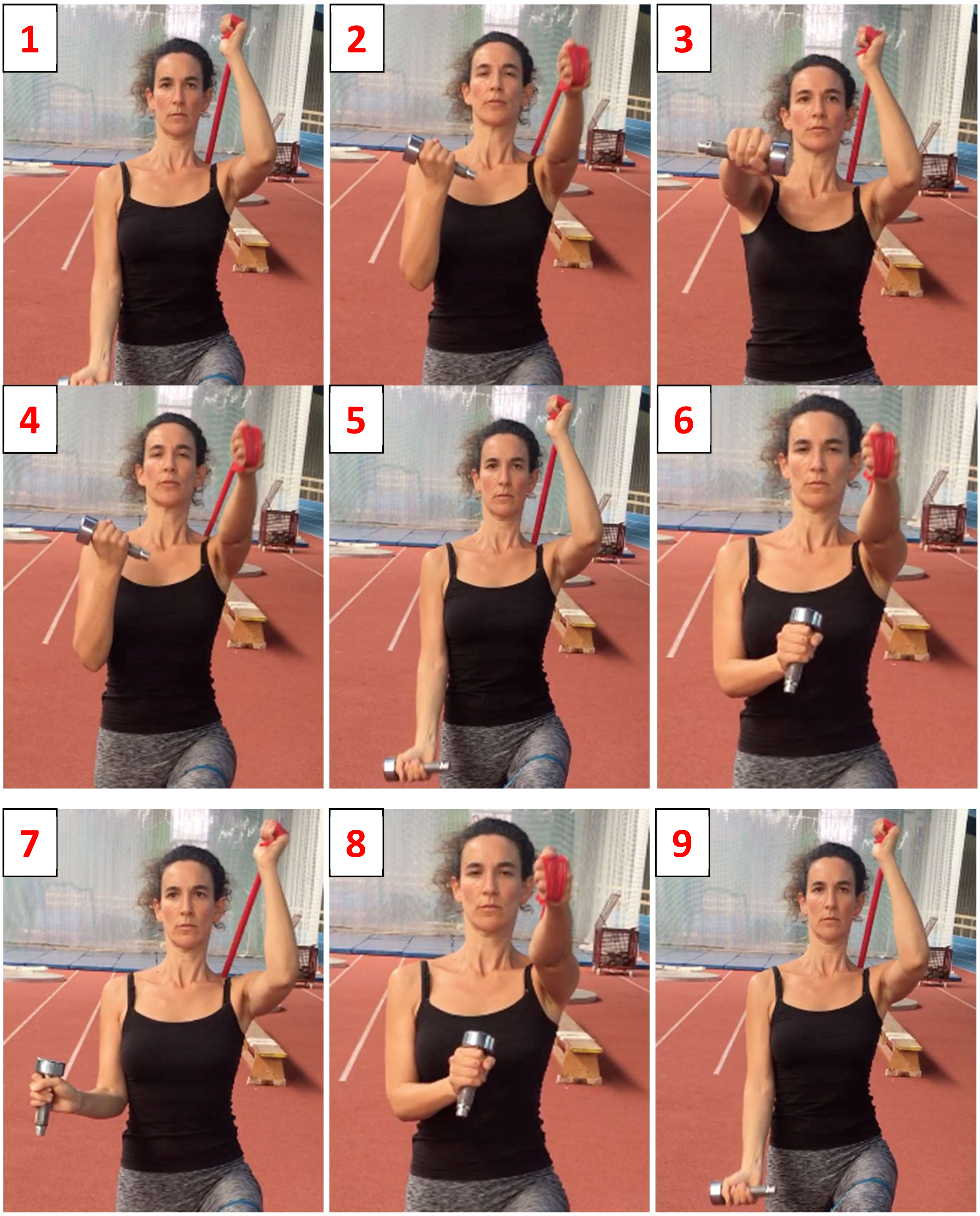

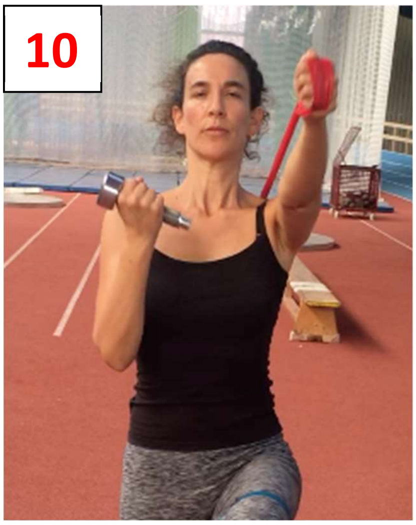
10
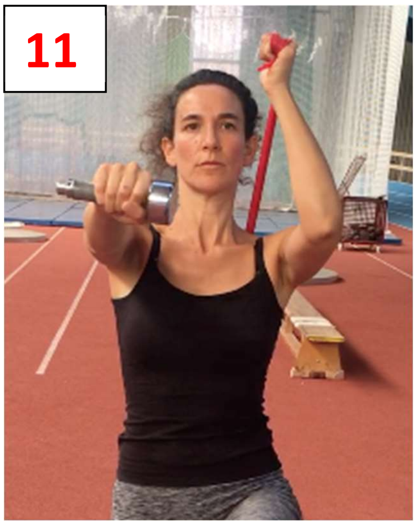
11
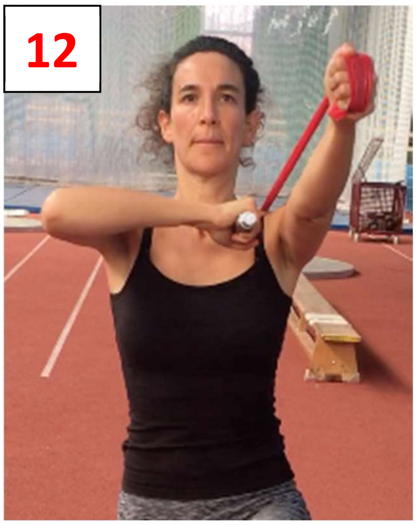
12
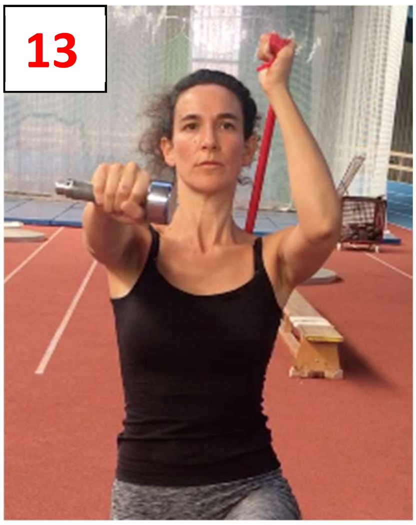
13
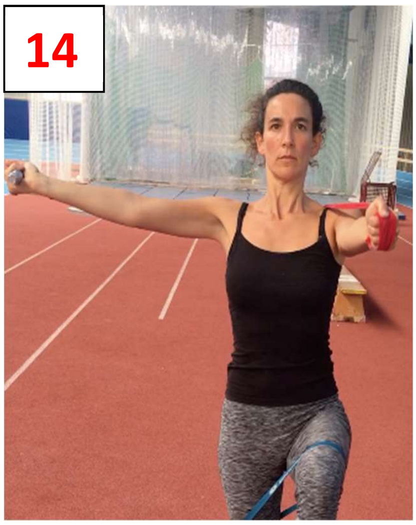
14
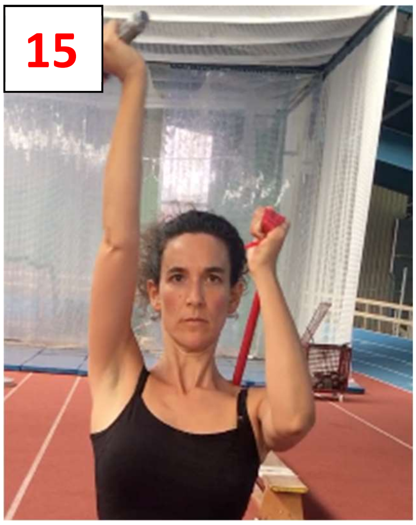
15
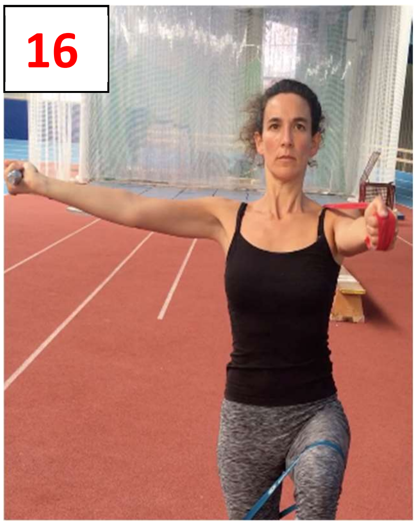
16
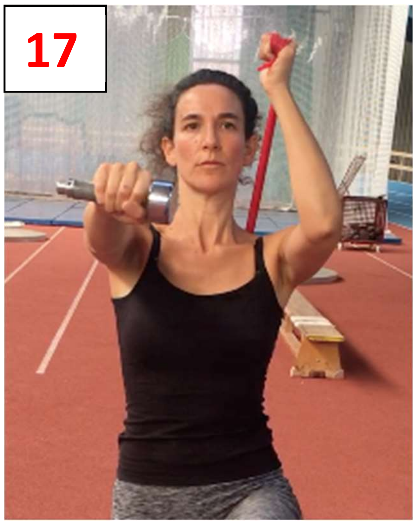
17
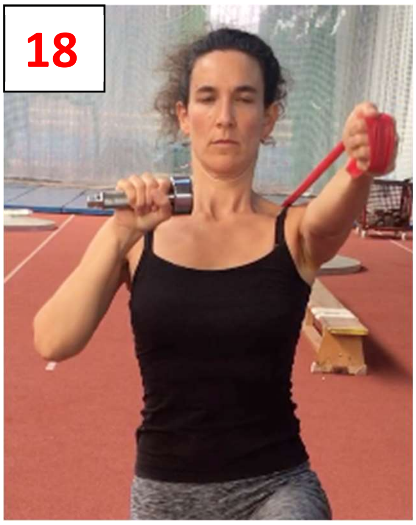
18
